# Supplementary material for: Efficient 3D light-sheet imaging of very large-scale optically cleared human brain and prostate tissue samples
Source: Commun Biol. 2023 Feb 13;6:170. doi: 10.1038/s42003-023-04536-4 (PMC9925784; doi:10.1038/s42003-023-04536-4)
Supplement: Supplementary file 1 — Supplemental Material [file 42003_2023_4536_MOESM1_ESM.pdf]

**Supplementary figure 1:** Title: Mosaic 4 acquisition of the entire surface of a prostatectomy section sample.

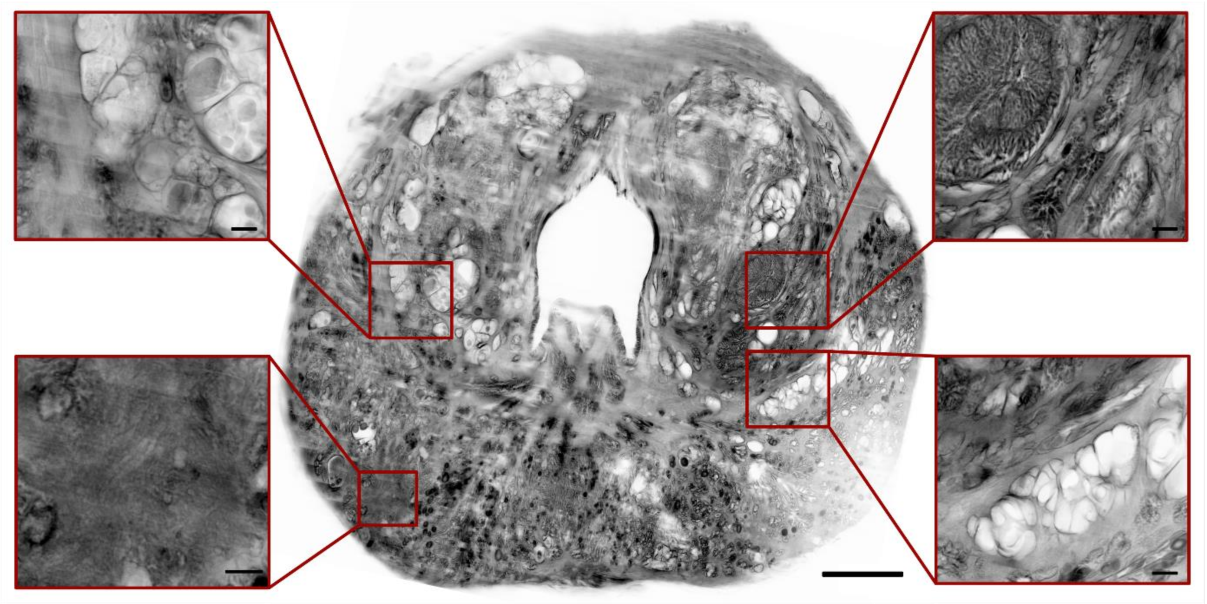

**Supplementary figure 1:** Legend: The image shows the stitched surface scan of whole prostate slice at 4 µm isotropic voxel size (middle), with 4 regions highlighted in the magnified inserts. Upper left and lower right: Area of a benign prostatic glandular hyperplasia, consisting of large dilated prostatic glands with intraluminal invaginations. Upper right: Normal prostatic glands of the transition zone consisting of middle large prostatic glands with intervening stroma. Lower left: Tumour region, which appears optically dense in the downsampled 4 µm data set. This is likely due to the many small round oval neoplastic glands with little intervening stroma and without intraluminal projections that make up the tumour, as was confirmed by histopathological evaluation of the corresponding H&E section. Scale bars: Whole sample scan: 5 mm; inserts: 500 µm, respectively.

**Supplementary figure 2:** Title: Changes of tumour morphology over depth in 3D.

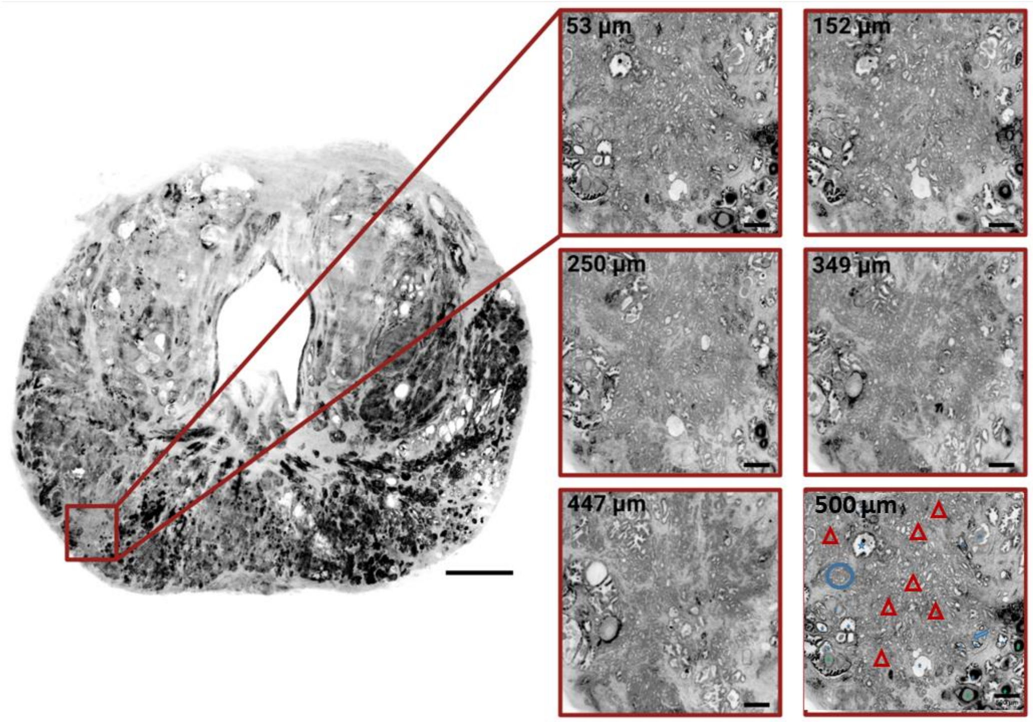


**Supplementary figure 2:** Legend: Left: 3D rendering of the entire prostate volume, with the tumour highlighted in red. Right: Tumour morphology at different depths throughout the data volume. The tumour consists of round oval neoplastic glands, compatible with the Gleason pattern 3. In deeper levels (500um) the neoplastic glands are packed very closed to each other, and fusion of some glands might be considered (blue circle), compatible with the higher grade of the prostate cancer grading system, namely Gleason pattern 4. Other area showed a focus of a small glands with almost inconspicuous lumen (blue arrow), morphologically might be compatible with the poorly formed glands of Gleason grade 4. Scale bars: 5 mm (left); 500 µm (right, all panels). Benign prostatic glands can be recognised by irregular gland surface and larger volume (blue stars), as compared with the neoplastic proliferation (red triangles). Corpora amylacea are easy identifiable in the benign glands (green stars).

**Supplementary figure 3:** Title: Tissue shrinkage after the MASH clearing protocol. a) Example of the surface area measurement before (top) and after (bottom) MASH clearing.

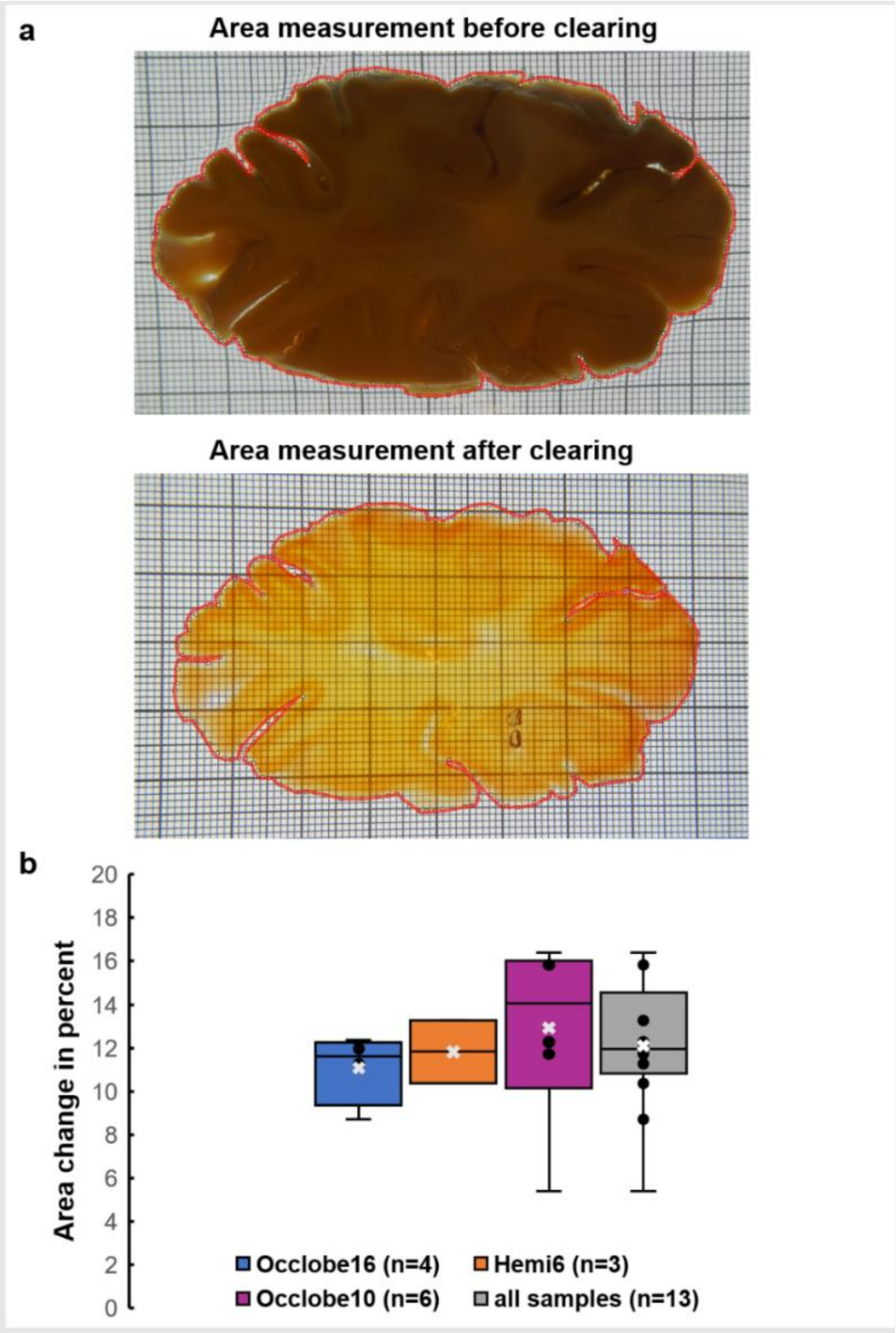

**Supplementary figure 3:** Legend: b) Shows box-and-whisker plots of the area change in percent for all 3 donors, as well as the pooled data from all 13 brain slices. Whiskers and box compartments show lower and upper quartiles, and interquartile range, respectively. The black horizontal line indicates the median and the white cross the mean of the data. Individual data points are shown as black dots, unless they would be located directly on the box or whisker lines.

**Supplementary figure 4:** Title: Camera and stage axes in ct-dSPIM imaging, and image viewer axes and their relationships.

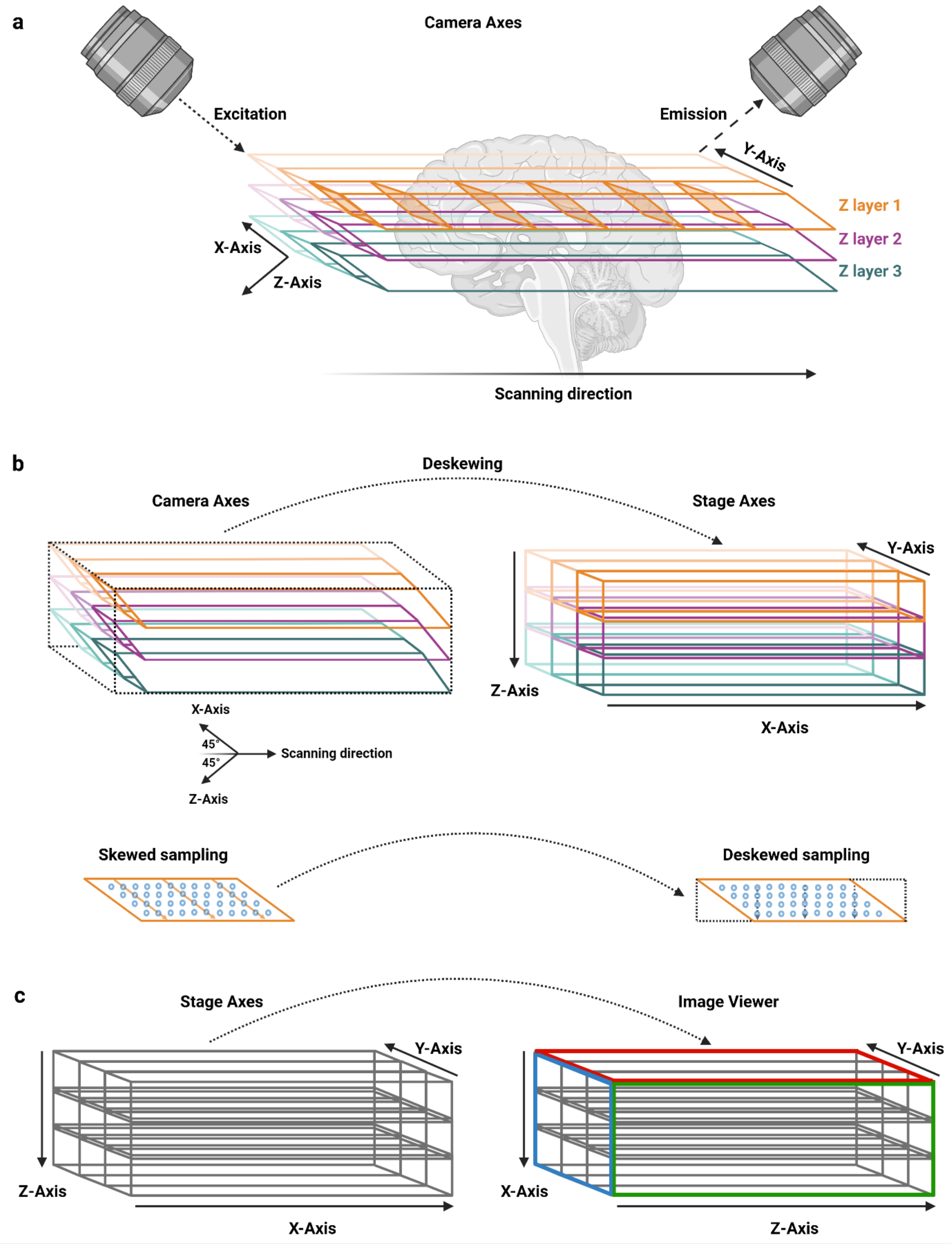

**Supplementary figure 4:** Legend: a) When acquiring image volumes with the ct-dSPIM, which uses stage scanning to move the sample through the imaging plane (i.e. the light sheet) the axes do not correspond to orthogonal XYZ coordinates. Instead, the camera Z-axis (perpendicular to the imaging plane) is at a 45° angle towards the scanning direction which is performed along the x-axis of the stage. Similarly, the camera X-axis is at a 45° angle towards the scanning direction. The Y-axis is the only axis that is shared between camera axes and stage axes. Therefore, stage-scanned image acquisition leads to a skewed parallelepipedal stack shape with non-orthogonal axes, which will be warped when viewed in the orthogonal axis system b) Shows how the deskewing operation transforms the skewed parallelepipedal stack (top, left), into a rectangular stack (top, right). If the stage step size along the scanning direction is chosen equivalent to the final, downsampled pixel size of a Mosaic acquisition (and therefore has a sqrt(2) relationship to the in-plane XY resolution in camera axes), the deskewing operation is equivalent a fast resampling of the skewed data, without the need for slow interpolation (bottom), although interpolation can be performed for every chosen stage step size c) As is the case for most 3D imaging data, image viewer software may use its own axis labelling which may be different from the that of the data imported into the viewer. The image viewer axis labeling here is the one used in figures in this work. Note that the deskewing step in b and axis relabeling step in c may be combined into the same processing step or reversed in order in actual software implementations.

**Supplementary figure 5:** Title: Manual validation of automated cell segmentation.

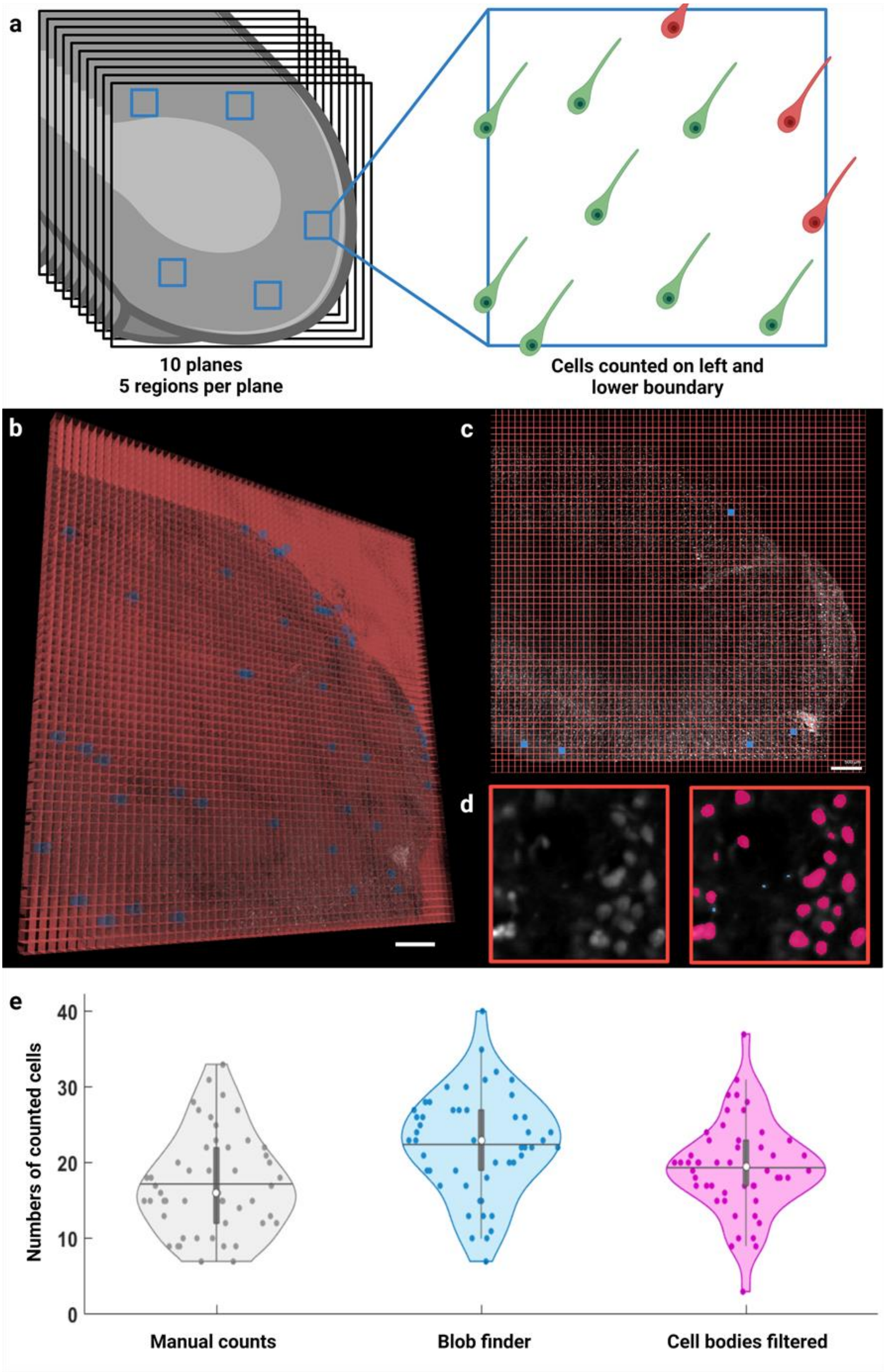

**Supplementary figure 5** Legend: a) Schematic representation of the validation procedure. 10 planes from the same dataset as shown in figure 7 were pseudo-randomly selected using a random number generator. On each plane, 5 ROIs (100x100 µm), selected in the same manner, were used for cell counting. Cells were counted that were either fully contained within the ROI or intersecting with the left and lower boundary. b) 3D rendering of the entire dataset, with the 100x100 µm grid overlaid in red. All ROIs that were counted on all 10 planes are depicted in blue. c) One representative plane out of the 10 selected ones, with the grid overlaid in red and the 5 ROIs displayed in blue. d) Single ROI showing the raw data (left) and the automatic segmentation results. The filtered segments, taking only object larger than 125 µm3 into account, are shown in magenta. The smaller segments which were detected by the "blob finder" segmentation of Vision4D, but have been filtered out, are shown in light blue. e) Violin plots for the manually counted cells (grey), the unfiltered segmentation results ("blob finder", blue) and the filtered segmentation results ("cell bodies filtered", magenta). White dots within the box plots indicate the median, the horizontal lines represent the mean for each dataset. Scale bars: 500 µm each.
