# Supplementary material for: Efficient 3D light-sheet imaging of very large-scale optically cleared human brain and prostate tissue samples
Source: Commun Biol. 2023 Feb 13;6:170. doi: 10.1038/s42003-023-04536-4 (PMC9925784; doi:10.1038/s42003-023-04536-4)
Supplement: Supplementary file 2 — Description of Additional Supplementary Files [file 42003_2023_4536_MOESM2_ESM.pdf]

Description of Additional Supplementary Files

File name: Supplementary Video 1

Description: Plane-by-plane view of the resliced Mosaic 16 scan of occipital lobe slice 1.

File name: Supplementary Video 2

Description: Plane-by-plane view of the resliced Mosaic 16 scan of occipital lobe slice 2. The sample is viewed along the YZ plane, to visualize its full extend. The volume is represented in inverted greyscale.

File name: Supplementary Video 3

Description: 3D rendering of an entire human occipital lobe slice 2 (3 mm thick). The slice was imaged with a Mosaic 16 scan. The mesoscopic and isotropic resolution of the dataset is sufficient to appreciate large anatomical landmarks and differences in the cytoarchitecture.

File name: Supplementary Video 4

Description: 3D rendering of the single view volume (human occiptital lobe) obtained at the highest resolution possible with the ct-dSPIM. ROIs (red spheres) were places manually along the volume, which was rotated and clipped to evaluate the placement of the ROIs within the layers.

File name: Supplementary Video 5

Description: Changes of prostate morphology in 3D. The video shows an average intensity projection of the prostatectomy slice (left, second 1-2), with normal prostatic glands of the transition zone highlighted in cyan and the tumor region highlighted in yellow (right), respectively. Second 4-17 show the change of prostate morphology as one moves plane-by-plane through the resliced data set. This allows to trace the changing shapes of both benign, as well as cancerous tissue elements.

File name: Supplementary Data 1

Description: Manual validation results

File name: Supplementary Data 2

Description: Shrinkage measurements results
